# Antitumor efficacy of the heparan sulfate mimic roneparstat (SST0001) against sarcoma models involves multi-target inhibition of receptor tyrosine kinases

**DOI:** 10.18632/oncotarget.10292

**Published:** 2016-06-25

**Authors:** Giuliana Cassinelli, Enrica Favini, Laura Dal Bo, Monica Tortoreto, Marcella De Maglie, Gianpaolo Dagrada, Silvana Pilotti, Franco Zunino, Nadia Zaffaroni, Cinzia Lanzi

**Affiliations:** ^1^ Molecular Pharmacology Unit, Department of Experimental Oncology and Molecular Medicine, Fondazione IRCCS Istituto Nazionale dei Tumori, Milan, Italy; ^2^ Department of Veterinary Sciences and Public Health, Università degli Studi di Milano, Milan, Italy; ^3^ Mouse and Animal Pathology Laboratory, Fondazione Filarete, Milan, Italy; ^4^ Laboratory of Experimental Molecular Pathology, Department of Diagnostic Pathology and Laboratory, Fondazione IRCCS Istituto Nazionale dei Tumori, Milan, Italy

**Keywords:** roneparstat, sarcoma, receptor tyrosine kinase, heparan sulfate, heparanase

## Abstract

The heparan sulfate (HS) mimic/heparanase inhibitor roneparstat (SST0001) shows antitumor activity in preclinical sarcoma models. We hypothesized that this 100% N-acetylated and glycol-split heparin could interfere with the functions of several receptor tyrosine kinases (RTK) coexpressed in sarcomas and activated by heparin-binding growth factors. Using a phospho-proteomic approach, we investigated the drug effects on RTK activation in human cell lines representative of different sarcoma subtypes. Inhibition of FGF, IGF, ERBB and PDGF receptors by the drug was biochemically and functionally validated. Roneparstat counteracted the autocrine loop induced by the *COL1A1/PDGFB* fusion oncogene, expressed in a human dermatofibrosarcoma protuberans primary culture and in NIH3T3^COL1A1/PDGFB^ transfectants, inhibiting cell anchorage-independent growth and invasion. In addition, roneparstat inhibited the activation of cell surface PDGFR and PDGFR-associated FAK, likely contributing to the reversion of NIH3T3^COL1A1/PDGFB^ cell transformed and pro-invasive phenotype. Biochemical and histological/immunohistochemical *ex vivo* analyses confirmed a reduced activation of ERBB4, EGFR, INSR, IGF1R, associated with apoptosis induction and angiogenesis inhibition in a drug-treated Ewing's sarcoma family tumor xenograft. The combination of roneparstat with irinotecan significantly improved the antitumor effect against A204 rhabdoid xenografts resulting in a high rate of complete responses and cures. These findings reveal that roneparstat exerts a multi-target inhibition of RTKs relevant in the pathobiology of different sarcoma subtypes. These effects, likely cooperating with heparanase inhibition, contribute to the antitumor efficacy of the drug. The study supports heparanase/HS axis targeting as a valuable approach in combination therapies of different sarcoma subtypes providing a preclinical rationale for clinical investigation.

## INTRODUCTION

Sarcomas are a highly heterogeneous group of rare aggressive tumors arising either in bones or soft tissues. Ewing's sarcoma (ES), rhabdomyosarcoma (RMS), osteosarcoma (OS) and synovial sarcoma (SS) are the most common forms in children and young adults [1, 2].

Although survival of sarcoma patients has improved in the last few decades, advanced and recurrent disease remains a challenge to clinical management and is associated with poor prognosis [1, 2]. In fact, current aggressive therapies with cytotoxic agents give low response rates in most histological subtypes and are associated with several side effects. The development of novel treatment approaches is needed to improve patients' outcomes [1]. Recent advances in elucidation of mechanisms of sarcoma molecular pathology have provided the ground to develop new molecularly targeted treatments based on abnormalities in growth factor signaling identified in the different sarcoma subgroups. Angiogenesis-related pathways are recognized as potential therapeutic targets and various agents targeting receptor tyrosine kinases (RTKs) are under clinical evaluation [3]. Imatinib, a TK inhibitor targeting ABL, KIT and PDGFR, has shown impressive efficacy in gastrointestinal stromal tumors carrying gain-of-function *KIT* or *PDGFRA* mutations, and in dermatofibrosarcoma protuberans (DFSP) characterized by overactivation of PDGFR due to a collagen 1A1 *(COL1A1)/PDGFB* rearrangement [4, 5]. Such therapeutic success, relying on a condition of ‘oncogene addiction’ [6], has not been reproduced in other sarcoma types. In fact, most of these tumors might not be dependent on a single targetable signaling pathway due to the high biomolecular complexity.

Growing preclinical and clinical evidence suggests that the heparanase/heparan sulfate (HS) system, a crucial regulator of biological processes in the tumor and its microenvironment, might represent a valuable therapeutic target [7–11]. HS, structurally similar to heparin, forms the side chains of HS proteoglycans (HSPGs) which are key components of the extracellular matrix (ECM) and the cell surface [10, 11]. HSPGs can exert structural and regulatory functions by contributing to the ECM integrity and by binding, through the docking-sites provided by the HS chains, a multitude of bioactive “heparin-binding” molecules including growth factors, cytokines and chemokines. This binding capability allows HSPGs to regulate the bioavailability and function of growth factors by creating a protected reservoir and by acting as co-receptors for ligands of RTKs [11]. HSs, are substrates for heparanase which is the only known mammalian endoglycosidase able to specifically cleave HS chains producing discrete fragments that facilitate the biological activity of bound (e.g. pro-angiogenic factors VEGF and bFGF). Moreover, heparanase enzymatic activity participates in ECM degradation and remodeling associated with processes involving cell dissemination, such as metastasis, inflammation, and angiogenesis. In fact, heparanase, which is rarely expressed in normal tissues, has been found highly expressed in several tumor types including RMSs and ESs, often associated with poor prognosis, and recently involved in chemoresistance [9, 12–15].

HS mimics, synthesized and selected as heparanase inhibitors, have shown anti-tumor efficacy as well as antiangiogenic and antimetastatic properties, in preclinical studies leading a few of them to clinical evaluation [9, 16]. We previously demonstrated the antitumor effect of the glycol-split heparin derivative heparanase inhibitor roneparstat (SST0001) in a panel of pediatric sarcoma models including an ES, RMSs, and OSs [13, 17]. Moreover, combination studies showed an improved treatment efficacy in association with clinically available antiangiogenic agents such as bevacizumab and sunitinib [17]. The nature of HS mimics suggests a complex mechanism of action affecting the plethora of functions of cellular and ECM-bound HS. Beyond heparanase, HS mimics are supposed to inhibit the function of heparin-binding molecules, including several growth factors of RTKs, and are likely to have an effect on cell signaling. Since, in most cases, such effects have only been assumed and not directly addressed, a better understanding of the multi-target actions of HS mimics on deregulated signaling pathways in specific tumor contexts is essential to optimize their use as antitumor drugs.

In the present study, we hypothesized that the activity of RTKs variably expressed and often over-active in sarcomas (e.g. FGF, ERBB, PDGF receptors) might be influenced by HS mimics. To test this hypothesis, we investigated the effects of roneparstat on critical signaling pathways and features of the malignant phenotype in sarcoma models.

## RESULTS

### Multi-target effects of roneparstat on RTK activation in pediatric sarcoma cell lines

**Figure 1:**
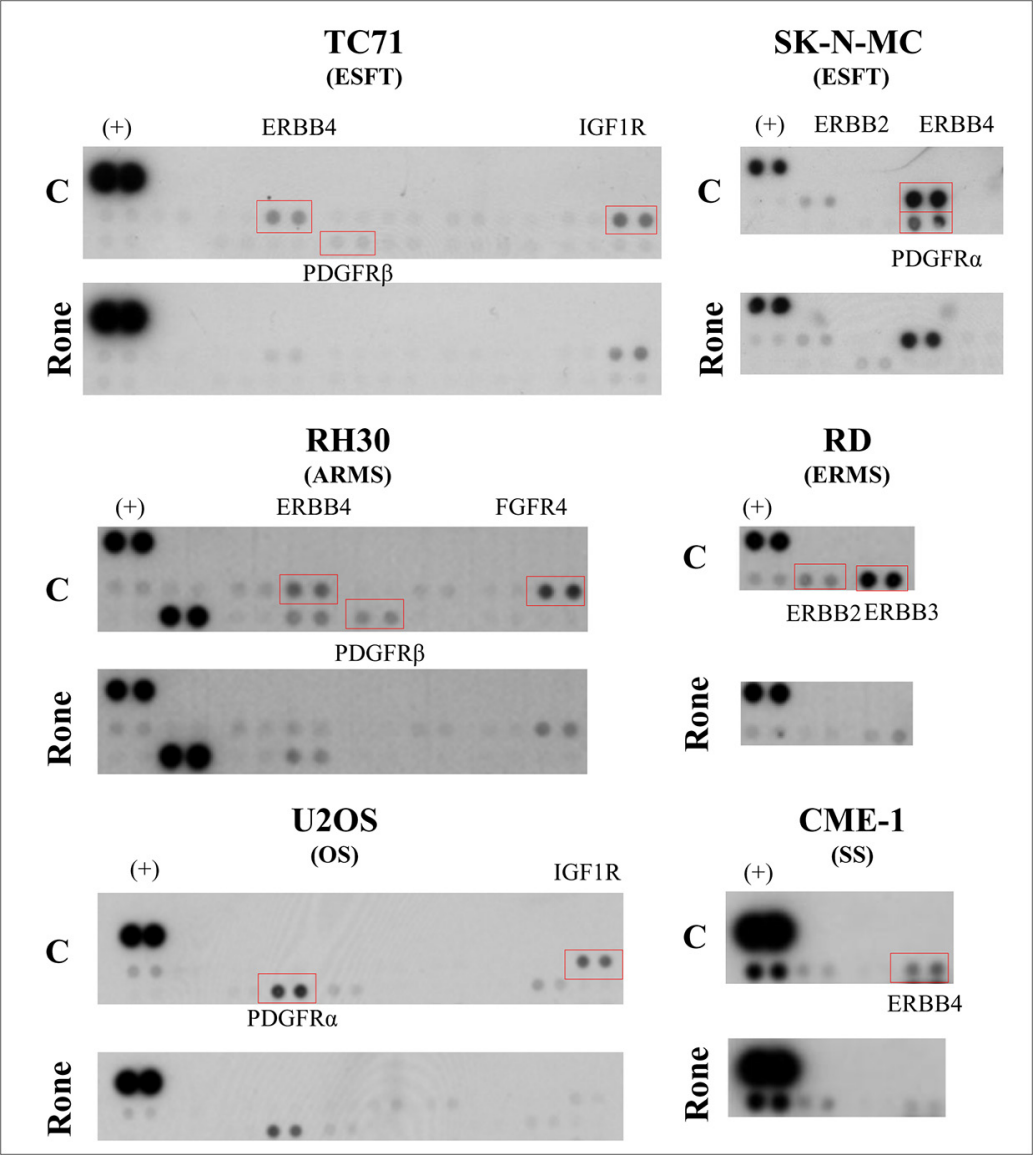
Proteomic profiling of tyrosine phosphorylated kinase receptors (RTKs) in control and roneparstat-treated sarcoma cell lines The day after seeding, cells were treated with solvent or roneparstat (1 mg/ml) for 48 h in complete medium. Then, control (C) and drug-treated (Rone) cells were lysed and processed for analysis with human phospho-RTK array. Rectangles evidence RTKs investigated in this study. (+), reference spots. ESFT, Ewing's sarcoma family tumor; ARMS, alveolar rhabdomyosarcoma; ERMS, embryonal rhabdomyosarcoma; OS, osteosarcoma; SS, synovial sarcoma.

We applied an explorative approach based on the phospho-proteomic profiling of RTKs to simultaneously detect the activation of multiple receptors in lysates from control and roneparstat-treated sarcoma cell lines including ES family tumors (ESFT), RMS, OS and SS. Whereas the inhibition of tyrosine phosphorylation of PDGFR and ERBB family members was a common event in roneparstat-treated cells from the different sarcoma histotypes, the drug interference on the activation of other receptors (i.e. IGF1R, FGFR4) was found to occur in a cell line-specific way (Figure 1). RTKs inhibited by roneparstat included receptors constitutively active in serum-free cultured cells, e.g. ERBB4 in ESFT and SS or FGFR4 in ARMS cells (Supplementary Figure S1), and receptors active in the presence of serum (Figure 1). Based on these findings, we sought to validate the HS mimic effects on receptor signaling pathways at biochemical and functional levels in selected sarcoma models.

### FGF/FGFR

**Figure 2:**
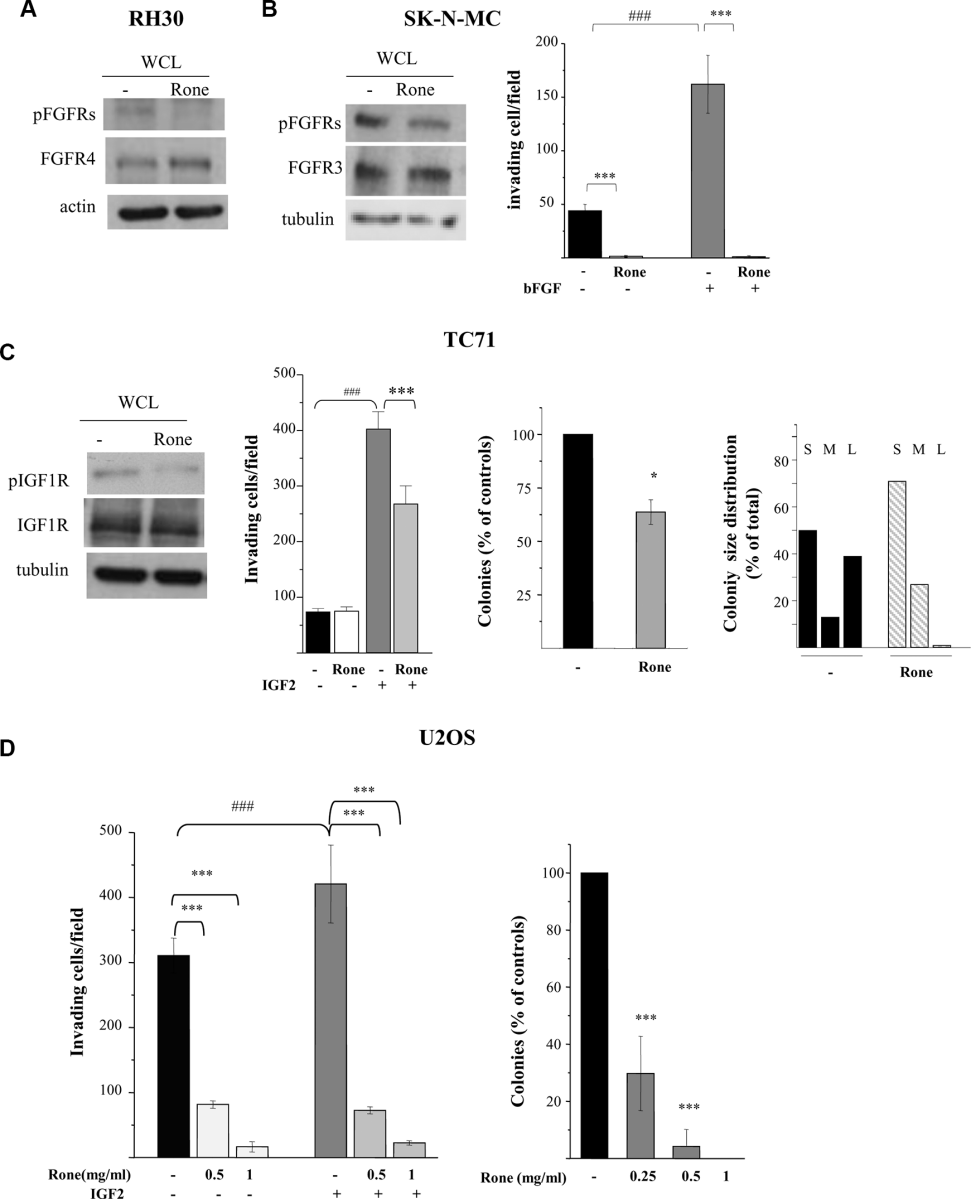
Inhibition of activation and biological activities of FGFRs and IGF1R (**A**), (**B**), (**C**) Western blot analyses were performed on whole cell lysates (WCL) from control (−) and roneparstat-treated (Rone) cells (1 mg/ml, for 48 h) to assess the receptor activation status using antibodies specifically recognizing activating tyrosine phosphorylated residues. The overall levels of receptors, actin or tubulin are shown as controls. (**B**), (**C**), (**D**) For the Matrigel invasion assay, cells were pretreated with roneparstat at 1 mg/ml or at the indicated concentrations for 24 h. Then, cells were transferred to Transwell chambers in serum-free medium with or without the indicated growth factors (50 ng/ml). The number of invading cells per field ± SD is reported. Data from one experiment representative of at least two independent experiments or the average data from two experiments, performed in independent duplicates, are shown. (**C**), (**D**) For the anchorage-independent cell growth assay, cells were seeded in soft agar in the presence or absence of roneparstat at 1 mg/ml (Rone) or at the indicated concentrations. U2OS cell colonies were counted after 24 days using a magnifying projector, whereas TC71 cell colony number and size were determined after 10 days by computer image analysis. The colony size distribution is scored as percentage of small (S, < 400 pixels), medium (M, 400–600 pixels), or large (L, > 600 pixels). Data from one experiment representative of at least two independent experiments, performed in duplicate (mean ± SD), are shown. *P < 0.05, ***P ≤ 0.001 drug-treated *versus* untreated control cells; ^###^*P* ≤ 0.001 growth factor stimulated *versus* unstimulated cells.

The inhibitory effect of drug treatment on FGFR4, constitutively active in RH30 cells (Supplementary Figure S1 and Figure 1), was confirmed by western blotting (Figure 2A). The activation of FGF receptors was not evidenced by the phospho-RTK array in the ESFT cell lines. However, since high levels of FGFR3 expression, associated with a cancer-related mutation, were described in SK-N-MC cells [18, 19], we analyzed tyrosine phosphorylation of this receptor by western blotting which confirmed the roneparstat-induced inhibition (Figure 2B). Moreover, bFGF-induced, as well as spontaneous, Matrigel invasion by SK-N-MC cells was abrogated by treatment with the HS mimic (Figure 2B).

### IGF/IGF-1R system

The phospho-RTK array analysis showed a reduced phosphorylation of IGF1R in roneparstat-treated TC71 and U2OS cells (Figure 1). Figure 2C shows that, in the ES cell line TC71, drug treatment was able to markedly reduce IGF1R phosphorylation at Y1135/Y1136, two major autophosphorylation sites in the receptor activation loop [20]. TC71 cells carry the prototypical EWS-FLI1 fusion and, consistently with a common pattern of IGF/IGF1R axis deregulation, express high levels of IGF1R and of both IGF1 and IGF2 likely involved in autocrine loops [21]. Nonetheless, IGF1R remains highly responsive to exogenous IGF2 in TC71 cells. In fact, as shown in Figure 2C, cell invasion in Matrigel was increased by 4-fold in the presence of the growth factor and such stimulatory effect was significantly antagonized by roneparstat. Treatment of the OS cell line U2OS, also producing the growth factor [22], inhibited in a dose-dependent way both spontaneous and exogenous IGF2-induced invasion (Figure 2D). Similarly, U2OS cell ability to form colonies in soft agar was significantly reduced by roneparstat and completely abrogated at 1 mg/ml (Figure 2D). Although this drug concentration modestly affected the overall clonogenic efficiency of TC71 cells (about 36% of inhibition), an almost complete disappearance of large-size colonies was observed (Figure 2C).

### ERBB family

**Figure 3:**
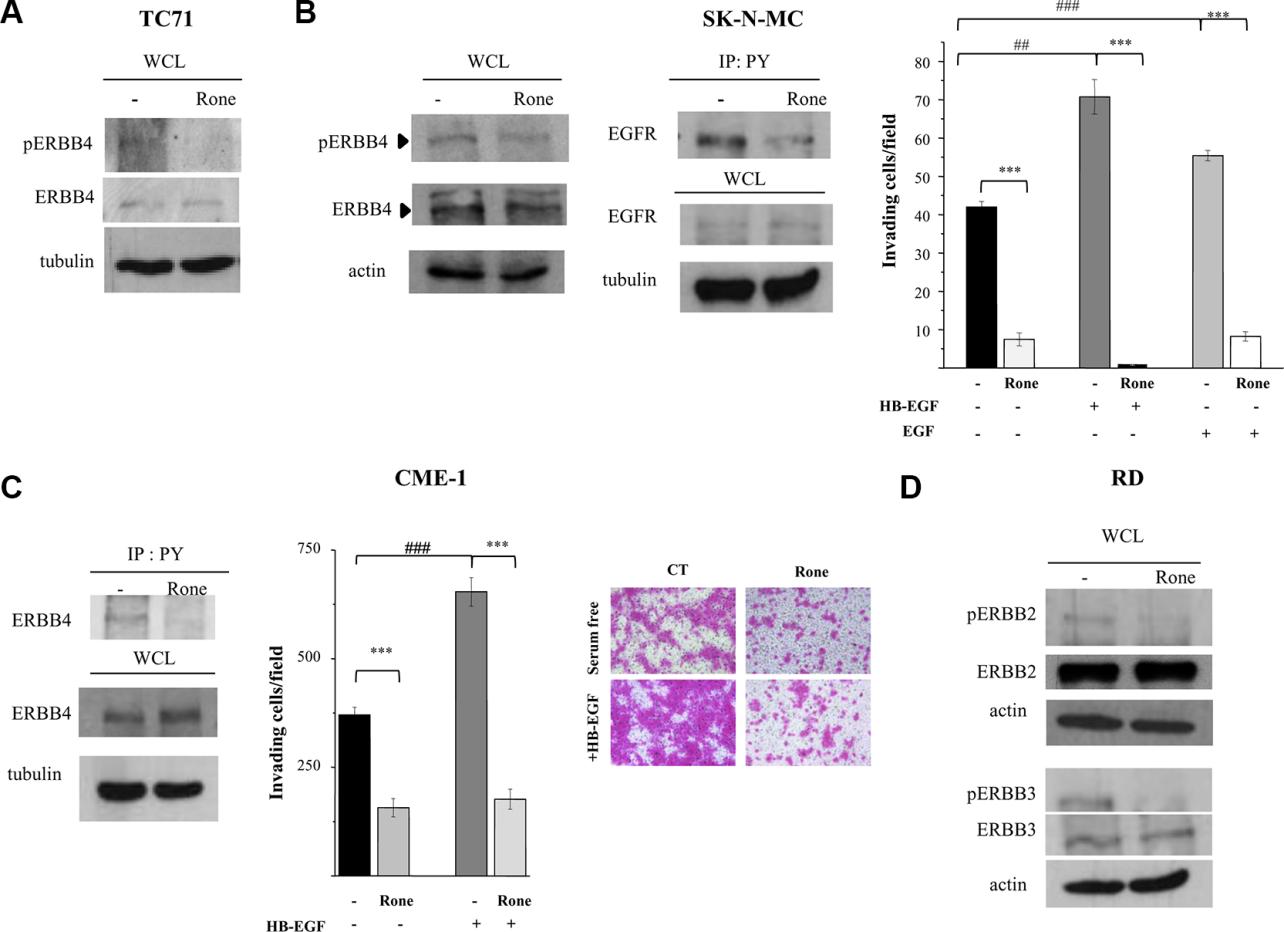
Inhibition of activation and biological activities of ERBB family receptors (**A**), (**B**), (**C**), (**D**) Western blot analyses were performed on whole cell lysates (WCL) from control (−) and roneparstat-treated cells (Rone, 1 mg/ml, for 48 h) to assess the receptor activation status using antibodies specifically recognizing kinase-activating phosphorylations. (B), (C) Activation of EGFR and ERBB4 was alternatively assessed by immunoprecipitation with anti-phospho-tyrosine antibody (IP: PY) followed by receptor detection by western blotting. The overall levels of receptors, actin or tubulin in the corresponding WCL are shown. Matrigel invasion assay was performed with cells previously exposed to 1 mg/ml roneparstat for 24 h in serum and then transferred in Transwell chambers in serum-free medium with or without the indicated growth factors (50 ng/ml). Data from one experiment representative of at least two independent ones (CME-1, mean ± SD) or the average data ± SE from two experiments (SK-N-MC), performed in independent duplicates, are shown. In (C), representative images show CME-1 cells passed through Matrigel and stained with SRB in the invasion assay, original magnification 100X. ***P ≤ 0.001 drug-treated versus untreated control cells; ^##^*P* ≤ 0.005 ^###^*P* ≤ 0.001 growth factor stimulated *versus* unstimulated cells.

Consistently with the phospho-proteomic findings (Figure 1), western blot analysis showed that roneparstat reduced the ERBB4 activating phosphorylation at tyrosine 984 in TC71 and SK-N-MC cell lines (Figure 3A and 3B) both deriving from post-chemotherapy ESFT and overexpressing a constitutively active receptor [19, 23–25 and Supplementary Figure S1]. Of note, a cancer-associated mutation of *ERBB4* has been described in TC71 cells [19]. ERBB4 can be activated by several members of the EGF-related growth factor family including heparin-binding-EGF (HB-EGF) characterized by a strong propensity to bind cell surface proteoglycans [26–28]. Accordingly, we observed that pretreatment of SK-N-MC cells with roneparstat clearly antagonized the HB-EGF stimulus in the Matrigel invasion assay. Moreover, under these conditions, drug treatment strongly inhibited also EGF-induced cell invasion (Figure 3B).

Since ERBB4 and EGFR, as homodimers or heterodimers, may share different ligands including HB-EGF and EGF [26], we further investigated the effects of roneparstat on EGFR activation. Despite EGFR was barely detectable in the phospho-RTK array (Figure 1) and in whole cell lysates (Figure 3B) of SK-N-MC cells, immunoprecipitation of tyrosine phosphorylated proteins from control and treated cells allowed demonstrating the ability of the HS mimic to inhibit the receptor phosphorylation (Figure 3B). Of note, roneparstat inhibited ERBB4 activation as well as HB-EGF-induced Matrigel invasion (Figure 3C) also in the CME-1 synovial sarcoma cell line, which harbors a constitutively active receptor (Supplementary Figure S1).

In line with the phospho-proteomic findings (Figure1), the activation of other ERBB family members, i.e ERBB2 and ERBB3, was found inhibited by roneparstat treatment in the ERMS cell line RD (Figure 3D) which expresses both receptors and a constitutively active ERBB3 [29, 30 and Supplementary Figure S1].

### PDGF/PDGFR

**Figure 4:**
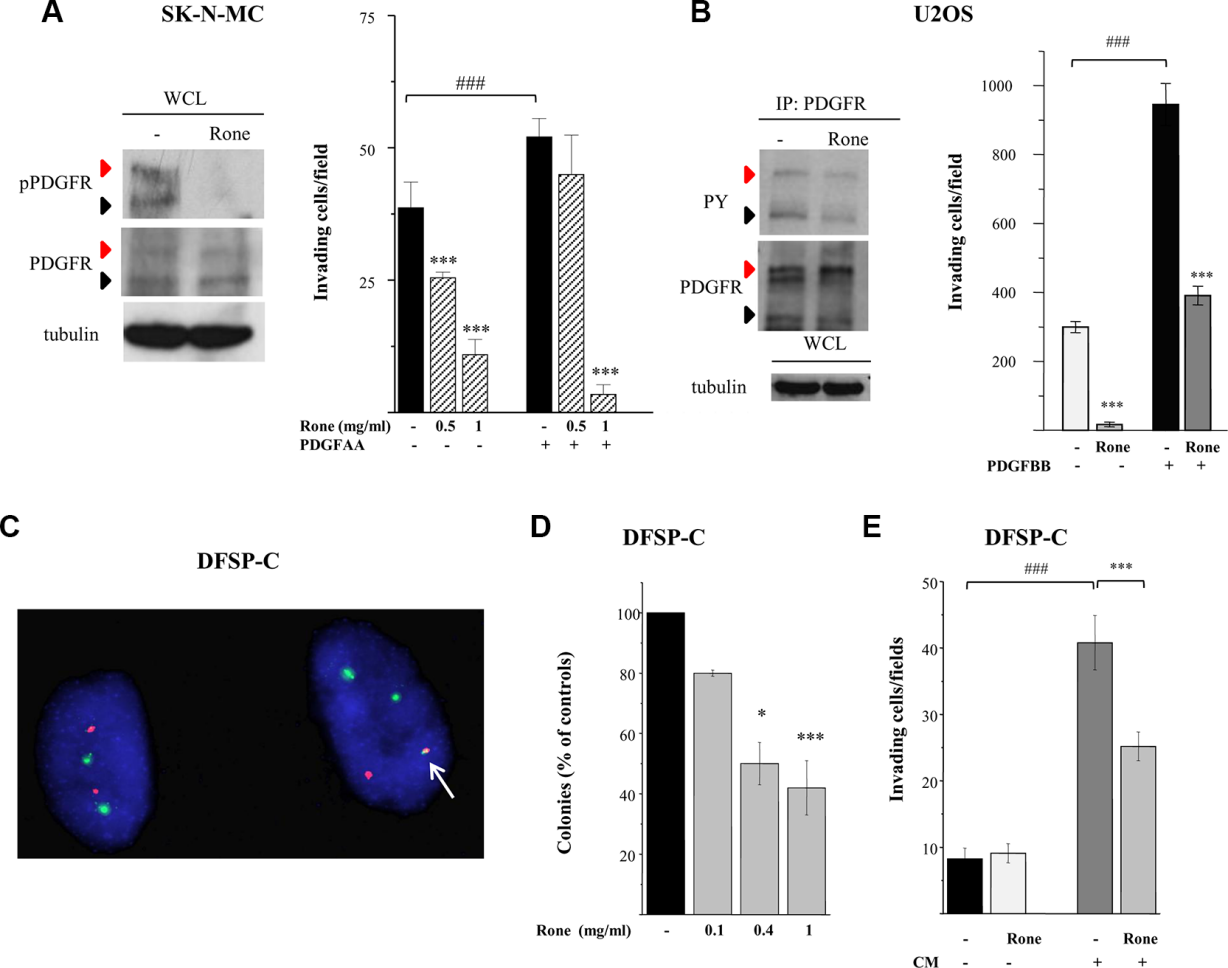
Inhibition of activation and biological activity of PDGFR receptors (**A**), (**B**) Western blot analysis was performed on whole cell lysates (WCL) or PDGFR immunoprecipitates (IP: PDGFR) from control (−) and roneparstat-treated cells (Rone, 1 mg/ml, for 48 h) with an antibody recognizing PDGFR activating tyrosines in SK-N-MC cells. Black arrows indicate the precursor form and red arrows the mature hyperglycosylated receptor. Tubulin shows correct loading. For the invasion assay, cells, pretreated with roneparstat at 1 mg/ml or at the indicated concentrations for 24 h, were transferred to Matrigel-coated transwells in serum-free medium in the presence of the indicated PDGFR ligands (50 ng/ml). In (A) and in (B), data from one experiment representative of at least two independent experiments, performed in duplicate, are shown. (**C**) The presence of the COL1A1/PDGFB fusion gene in DFSP-C short term cell culture was assayed by FISH analysis using green-labeled COL1A1 and spectrum orange-labeled PDGFB BAC probes. A single fusion signal (arrow) is present in the tumor cell harboring the DFSP specific translocation alongside with two green signals (COL1A1) and one red signal. On the left, a cell showing normal FISH pattern. (**D**) Inhibition of DFSP-C cell anchorage-independent growth by roneparstat. Cells were seeded in soft agar in the presence or absence of increasing drug concentrations. Colonies were counted after 26 days using a magnifying projector and data reported as mean percentage of controls ± SD. (**E**) Inhibition of Matrigel invasion. DFSP-C cells were subjected to invasion assay in serum-free medium, or in the presence of their own conditioned medium (CM), after 24 h of treatment with roneparstat (1 mg/ml). In (D) and (E) average data from two experiments, performed in independent duplicates, are shown. *P < 0.05, ***P ≤ 0.001 drug-treated versus untreated control cells, ^###^*P* < 0.001 growth factor stimulated versus unstimulated cells.

We previously reported the abrogation of PDGFR tyrosine phosphorylation in TC71 cells exposed to roneparstat [17]. Here, we deepened investigation of the drug effects on the PDGF/PDGFR axis in an additional ESFT (SK-N-MC) and in an OS (U2OS) cellular model. Differently from TC71 cells, displaying a barely detectable phosphorylation of PDGFRβ in the phospho-RTK array, SK-N-MC cells showed a marked activation of PDGFRα in the presence of serum which was completely inhibited in drug-treated cells (Figure 1). Western blotting of SK-N-MC cell lysates confirmed the abrogation of phosphorylation at tyrosine residue 849, the major autophosphorylation site in the receptor activation loop, which was evident in both the precursor and the mature forms [31] (Figure 4A). Moreover, a remarkable dose-dependent inhibition on PDGF-induced Matrigel invasion was observed in roneparstat-treated cells. The phospho-RTK array confirmed a constitutive activation of PDGFRα in U2OS cells (Supplementary Figure S1) accordingly to the described PDGF-mediated autocrine loop [31]. The reduced receptor phosphorylation observed following roneparstat-treatment (Figure 1) was further validated by immunoprecipitation (Figure 4B). Moreover, the drug strongly inhibited U2OS cell spontaneous invasiveness and abrogated the 3-fold increase in cell invasion induced by exogenous PDGF (Figure 4B).

### COL1A1/PDGFB/PDGFR oncogenic loop

The striking effects of roneparstat in sarcoma cells endowed with PDGFR activating autocrine loops [31 and our data not shown] prompted us to investigate the ability of the HS mimic to inhibit the transforming potential of the COL1A1/PDGFB chimeric protein generated by the chromosomal translocation t(17;22) in DFSP [4, 5]. In this tumor, the fusion protein is processed into a functional PDGFBB leading to an autocrine activation of PDGFRβ which is recognized as driver of the oncogenic transformation. We took advantage of the availability of an early passage human DFSP primary cell culture (DFSP-C) to investigate the effects of roneparstat on malignant phenotype features such as anchorage–independent growth and invasiveness. Fluorescent In Situ Hybridization (FISH) analysis confirmed the presence of the *COL1A1/PDGFB* fusion gene in about 85% of cells. The FISH pattern of a representative DFSP-C cell shown in Figure 4C, characterized by a single copy of the *COL1A1/PDGFB* fusion, is superimposable with that observed in the patient DFSP specimen (not shown). DFSP-C cell colony formation in soft agar was significantly inhibited in the presence of the HS mimic (Figure 4D). In the Matrigel invasion assay, DFSP-C cells showed a low spontaneous invasive potential, however, when incubated in the presence of their own conditioned medium, the invasive capacity was enhanced. Under this condition, a significant reduction of cell invasion could be observed upon roneparstat treatment (Figure 4E).

**Figure 5:**
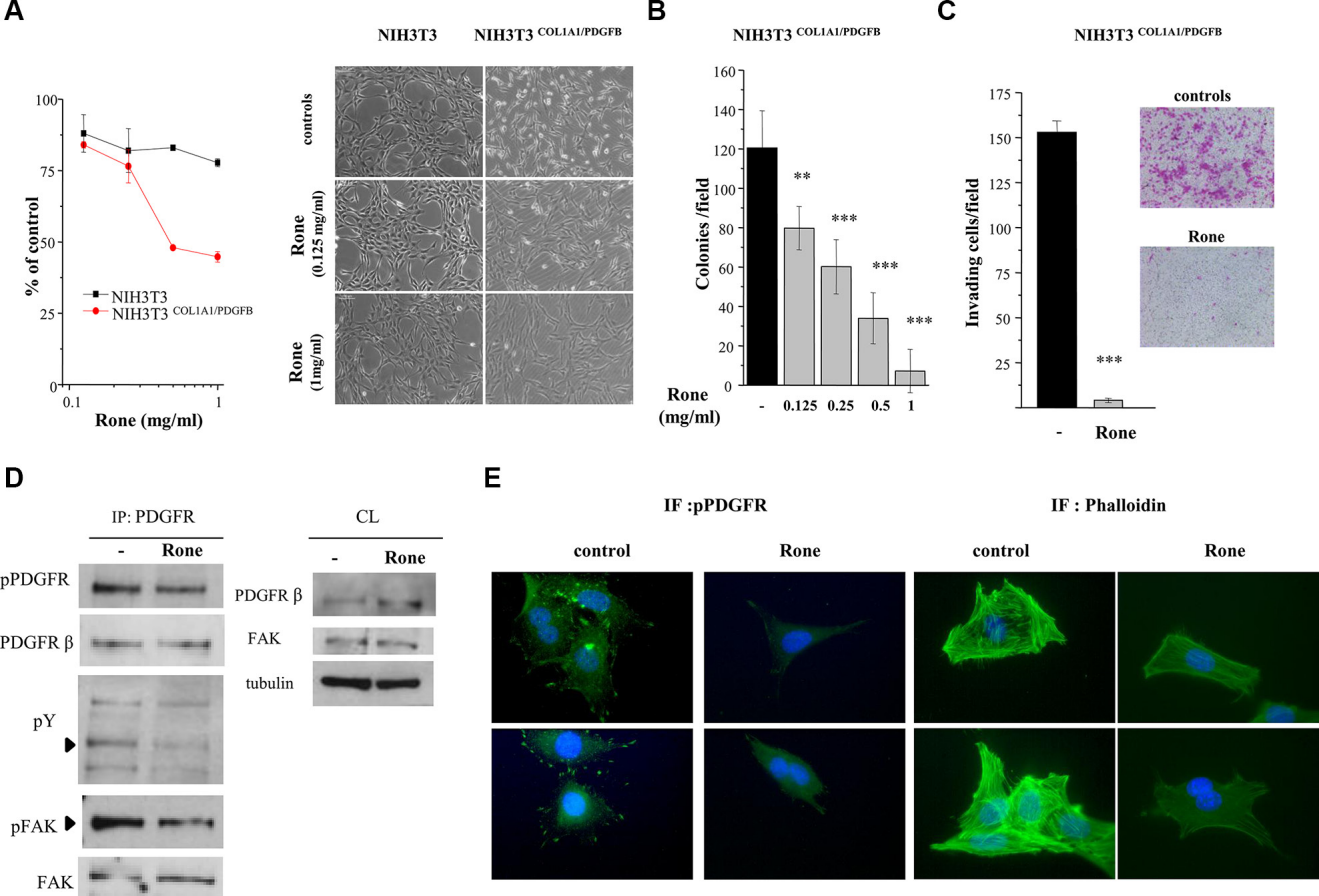
Inhibition of *COL1A1/PDGFB* fusion oncogene-mediated PDGFR activation and NIH3T3 cell transformation (**A**) Parental NIH3T3 and NIH3T3^COL1A1/PDGFB^ cells were treated with the indicated concentrations of roneparstat (Rone) and the drug antiproliferative activity was assessed 72 h later by cell counting. The right panel shows the effect of 24 h drug treatment on the transformed morphologic phenotype of NIH3T3^COL1A1/PDGFB^ cells in comparison with parental cells. Representative images were taken under a phase-contrast microscope (original magnification, 100X). (**B**) Inhibition of anchorage-independent growth of NIH3T3^COL1A1/PDGFB^ cells. Cells were seeded in soft agar in the presence or absence of increasing roneparstat concentrations. Colonies were counted after 11 days using a magnifying projector and data reported as mean colony number/field ± SD. (**C**) Inhibition of NIH3T3^COL1A1/PDGFB^ cell invasive ability. After 24 h of exposure to roneparstat (1 mg/ml), transfected fibroblasts were transferred to Matrigel-coated transwell chambers in serum-free medium and invasion assessed 24 h later. Data are reported as the average cell number per field ± SD. Representative images of SRB-stained invaded cells are shown beside (original magnification 100X). (**D**) Effect of roneparstat (1 mg/ml, 24 h) on PDGFR activation and signaling. The receptor was immunoprecipitated from NIH3T3^COL1A/PDGFB^ cell lysates with an anti-PDGFRβ antibody and its activation assessed by western blotting using an antibody recognizing tyrosine phosphorylated PDGFR. In the same filter, anti-phosphotyrosine antibody (pY) revealed phosphopeptides co-immunoprecipitated with PDGFRβ, among which FAK, which was then identified by blotting with anti-phospho-FAK and anti-FAK antibodies. Blots performed on cell lysates (CL) show the protein overall levels and loading control. (**E**) Indirect immunofluorescence showing, on the left, localization of tyrosine phosphorylated PDGFR in control and roneparstat-treated (1 mg/ml for 24 h) NIH3T3^COL1A/PDGFB^ cells. On the right, cellular distribution of F-actin stained with green fluorescent phalloidin. Nuclei are evidenced with Hoechst 3341 counterstaining (blue). Two images for each sample are shown. Original magnification, 1000X. **P < 0.01, ***P < 0.001 drug-treated versus untreated control cells. Data from representative experiments, performed in duplicate, are shown.

Further investigations were performed using NIH3T3 mouse fibroblasts transformed by human DNA containing the *COL1A1/PDGFB* rearrangement (NIH3T3^COL1A1/PDGFB)^ [5] as a DFSP model system. Roneparstat selectively inhibited the proliferation of NIH3T3^COL1A1/PDGFB^ with respect to parental NIH3T3 cells (Figure 5A). In addition, the drug completely reverted the transformed phenotype of transfected cells to a flattened, less refractile, normal fibroblast-like morphology characterized by contact inhibition, resembling that of parental NIH3T3 cells. Anchorage–independent growth, a hallmark of malignant cells, was also efficiently inhibited in a dose-dependent way (Figure 5B). Furthermore, the drug strongly reduced the invasive ability of NIH3T3^COL1A1/PDGFB^ cells under serum free-condition (Figure 5C). Western blot analysis of PDGFR immunoprecipitates from control and roneparstat-treated NIH3T3^COL1A1/PDGFB^ cells showed that the drug was able to reduce PDGFRβ phosphorylation at Y857 (Figure 5D), a residue located in the receptor activation loop and critical for TK activity [32]. Immunofluorescence microscopy showed that in NIH3T3^COL1A1/PDGFB^ control cells, the activated receptor was present either in a cytoplasmic pool, surrounding the nuclei, or at the cell periphery where it was concentrated in the filipodial rod-like extensions at the leading edge of polarized motile cells (Figure 5E). Such a subcellular localization of the activated PDGFR resembled the transient effect described for exogenous PDGFBB on the receptor distribution in fibroblasts [33]. In roneparstat-treated NIH3T3^COL1A1/PDGFB^ cells, the receptor activation was no more detected in the filopodial extensions, suggesting a specific effect on the PDGFR pool localized at the cell membrane. Indeed, western blot showed increased levels of PDGFRβ in drug-treated whole cell lysates (Figure 5D) consistent with interference on ligand-induced receptor downregulation [34]. Because of the well-known ability of the PDGFBB/PDGFRβ axis to induce cytoskeleton remodeling in mesenchymal cells [31], we examined the F-actin organization in our model. Consistent with the reduced cell motility of drug-treated NIH3T3^COL1A1/PDGFB^ cells (Figure 5C), F-actin staining by phalloidin evidenced the prevalent organization of actin filaments in not contractile cortical fibers. Conversely, in control cells the complex network of ventral stress fibers and transverse arcs reflected highly functional contractile machinery [33, 35].

In accordance with downregulation of PDGFR kinase activity in drug-treated NIH3T3^COL1A1/PDGFB^ cells, the tyrosine phosphorylation of co-immunoprecipitated proteins appeared reduced (Figure 5D). Since focal adhesion kinase (FAK) is known to link the growth factor/RTK systems with the ECM/integrins axes and to be associated with PDGFBB-activated PDGFRβ [36], we assessed the presence of FAK in PDGFRβ immunoprecipitates from NIH3T3^COL1A1/PDGFB^ cells. Although FAK was found associated with the RTK in both control and drug-treated cells (Figure 5D), its phosphorylation at tyrosine 397, located in the kinase domain, was markedly reduced in cells exposed to roneparstat. Overall, these findings demonstrated the ability of the HS mimic to inhibit PDGFRβ and its signaling activated through the pathogenic autocrine loop active in DFSP.

### Multi-RTK inhibitory effects of roneparstat *in vivo*

**Figure 6:**
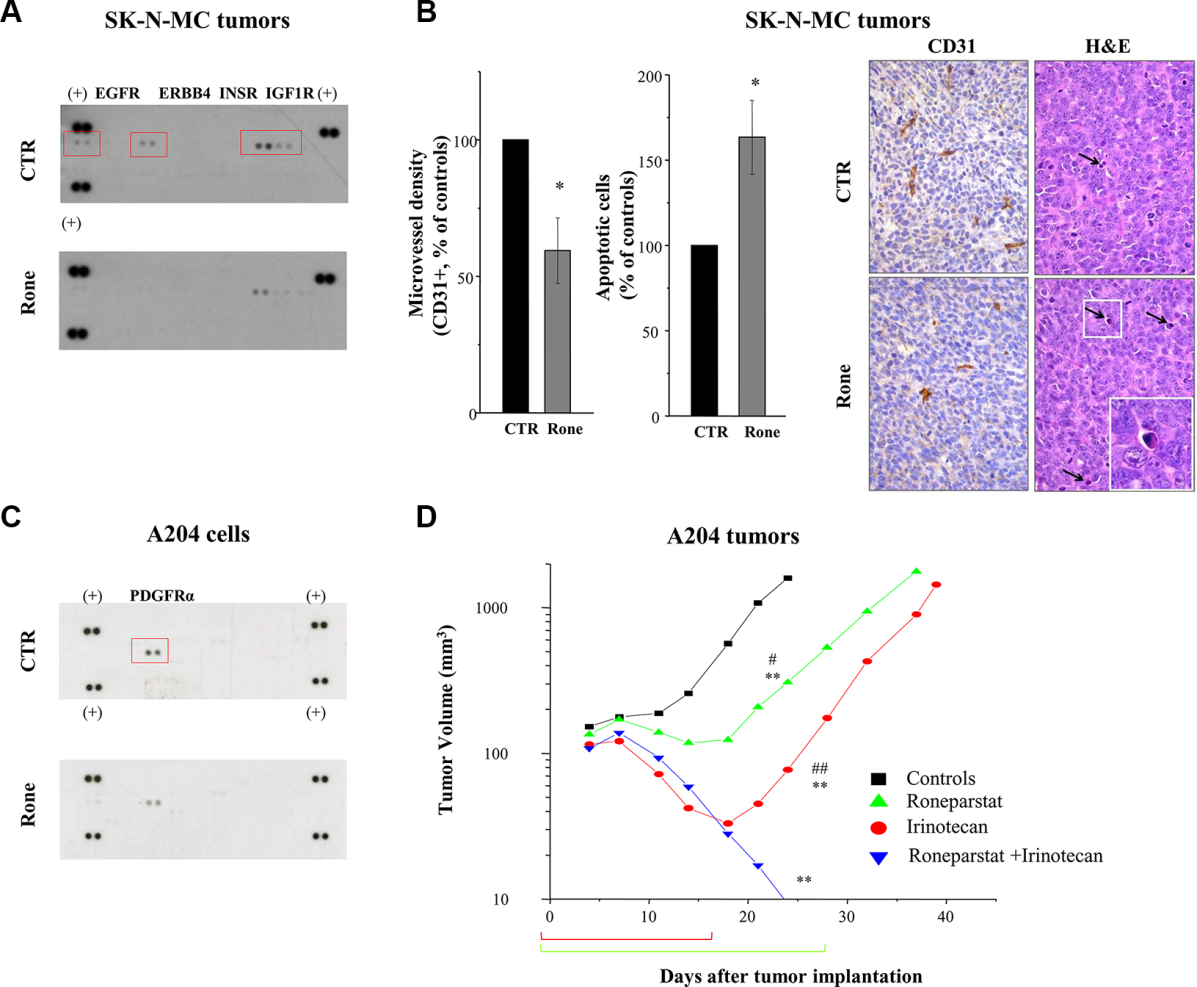
*In vivo* activity of roneparstat, alone and in combination, against sarcoma xenografts in mice (**A**), (**B**) Pharmacodynamic effect on RTKs in SK-N-MC xenografts associated with angiogenesis inhibition and apoptosis induction. Tumor xenografts-bearing mice were administered with vehicle (CTR) or roneparstat at 60 mg/kg (2qdx5/w)(Rone). After 16 days, tumors were removed and processed for proteomic profiling with phospho-RTK array (A) or immunohistochemical and histological analysis (B). For immunohistochemical detection of microvessel density, formalin fixed and paraffin embedded tumor sections were probed with an antibody recognizing CD31-positive cells. In parallel, tumor sections were stained with Hematoxylin-Eosin (H & E) for morphological detection of apoptotic cells. Columns, mean percentage of controls ± SE. On the right, representative images of CD31 and H&E staining. Arrows indicate apoptotic cells with typical morphological features: shrinkage and fragmentation into membrane-bound apoptotic bodies. Original magnification 400X; insert, 1000X. (**C**) Reduced constitutive phosphorylation of PDGFRα in roneparstat-treated A204 rhabdoid cells. After 48 h of incubation in the presence of solvent or 1 mg/ml roneparstat in serum-free medium, cells were lysed and processed for proteomic profiling with phospho-RTK array. (+), reference spots. (**D**) Enhancement of antitumor efficacy against A204 rhabdoid xenografts by combined treatment with irinotecan. Irinotecan (50 mg/kg) was administered i.v. with an intermittent treatment schedule q4dx4; roneparstat (60 mg/kg) was administered s.c., 2qdx6/w, for 4 weeks. Brackets under abscissa indicate the treatments' timeframe. *P < 0.05, **P < 0.01, drug-treated versus control tumors, ^#^*P* < 0.05, ^##^*P* < 0.01 versus drug combination at day 24 after tumor implantation.

Using the previously reported treatment schedule (s.c., 2qdx6/w x4w) [13, 17], here we extended the analysis of roneparstat activity on an additional human ESFT model, SK-N-MC. Moreover, we investigated the drug potential to affect RTK activation in the *in vivo* setting. SK-N-MC xenografts were responsive to roneparstat treatment which induced a maximum TVI of 67% (*P* < 0.003) (day 17 after the beginning of treatment) and 1/7 cured mice at the end of the experiment (day 117). Pharmacodynamic confirmation of roneparstat multitarget effect was obtained on SK-N-MC tumors excised after 12 days of treatment. Analysis of tumor tissue lysates by phospho-RTK array showed a prominent tyrosine phosphorylation of EGFR, ERBB4, INSR and IGF1R in control which was remarkably decreased in tumor from treated mice (Figure 6A). In addition, immunohistochemical detection of CD31 and histological analyses showed that roneparstat induced a reduction of microvessel density (*P* < 0.05) and an increase of the apoptotic nuclei number (*P* < 0.05) (Figure 6B), whereas the number of mitoses in tumor cells was not significantly affected (not shown). Increased apoptosis and decreased angiogenesis were also observed in archived tissue samples of RD tumors from mice which received prolonged (3–4 weeks) roneparstat treatments [17] (Supplementary Figure S2). Interestingly, in this model, a significant reduction of mitoses in tumor cells could be also observed.

### Enhanced antitumor effect by combination of roneparstat with irinotecan

We previously showed that angiogenesis inhibition contributes to the antitumor efficacy of camptothecins and found that roneparstat cooperates with antiangiogenic agents to counteract sarcoma xenograft growth [17, 37]. Thereby, we tested the potential of the HS mimic to promote responsiveness to irinotecan of the rhabdoid A204 tumor that displayed the lowest sensitivity to treatment with camptothecins, among a panel of sarcoma models, in terms of cures [37]. As reported in Figure 6C, roneparstat was able to decrease the constitutive activation of PDGFRα in A204 cells [38, 39]. Confirming previous findings [17, 37], roneparstat and irinotecan as single agents achieved a high TVI (80% and 95%, respectively) with one complete regression (CR) in the group of animals receiving the camptothecin (Table 1 and Figure 6D). Nonetheless, single drug administration produced mostly a tumor growth delay (Figure 6D). In mice receiving the drug combination, TVI reached 100%, with 8/8 animals experiencing CR. In this group of mice, only 3 tumors regrew around 50 days after the last treatment, whereas the remaining 5 out of 8 mice showed no evidence of disease at the end of the experiment (day 115) (Table 1). Noteworthy, the combination treatment was well tolerated in all animals.

**Table 1:** Antitumor effects of roneparstat and irinotecan against human A204 rhabdoid sarcoma xenografts

Drug	N^a^	Dose^b^ (mg/kg/day)	Schedule	TVI%^c^ (day)	CR^d^	NED^e^
**irinotecan**	8	50	q4dx4	95 (24)**,^##^	1/8	0/8
**roneparstat**	7	60×2	2qdx6/wx4w	80 (24)**,^#^	0/7	0/7
**roneparstat plus irinotecan**	8	60×2 50	2qdx6/wx4w q4dx4	100 (24)**	8/8	5/8

a N, number of treated mice

b Tumor fragments were implanted s.c. in the right flank of nude mice at day 0 and treatments started the day after. Roneparstat, dissolved in sterile saline, was administered s.c. at 10 ml/kg; irinotecan, dissolved in sterile distilled water, was delivered i.v. at 15 ml/kg. Drugs were administered, as indicated, alone or in combination.

c TVI%, tumor volume inhibition percent = 100 − (mean tumor volume of treated mice / mean tumor volume of control mice × 100) determined on day 24 after tumor implantation.

d CR, complete regressions, i.e. disappearance of the tumors lasting at least ten days after the end of treatments

e NED, mice with no evidence of disease at the end of the experiment (day 115).

** P < 0.001 vs control tumors

# P < 0.05

## P < 0.01 vs combination-treated tumors, by Student's *t* test.

## DISCUSSION

Consistent with the pleiotropic nature and the broad regulatory functions of HS, the mechanism of the antitumor action of HS mimics appears complex and dependent on the biological context. We previously reported a significant activity of the HS mimic roneparstat against a panel of soft tissue and bone sarcoma models [13, 17]. In the present study, we extended investigation to the effects exerted by the drug on molecular signaling systems implicated in the pathobiology of sarcomas. Our data showed that, by acting as a multi-target agent, roneparstat was able to counteract activation and functions of several growth factor/RTK axes supporting or driving the malignant phenotype in different sarcoma cell subtypes. The inhibition of specific RTKs was confirmed *ex vivo* in drug-treated ESFT xenografts. In addition, we showed that the combination of roneparstat with irinotecan, a clinically available cytotoxic agent, resulted in strong potentiation of the anti-tumor effect.

Roneparstat was selected in extensive synthetic chemistry studies as a modified heparin devoid of any significant anticoagulant effect and endowed with a strong heparanase inhibitory activity [40]. For its relevance in critical aspects of cancer progression, heparanase represents an attractive therapeutic target [7, 9, 16]. Indeed, inhibition of secreted heparanase, implicated in ECM remodeling processes, is consistent with the anti-angiogenic and anti-metastatic effects described for roneparstat and other HS mimics [7, 41–43]. Furthermore, the ability of roneparstat to inhibit multiple myeloma growth and angiogenesis has been correlated with disruption of the heparanase/syndecan-1 axis and downregulation of HGF, VEGF and MMP-9 gene expression controlled by endogenous heparanase in myeloma cells [44, 45]. In line with these findings, roneparstat treatment induced a remarkable reduction of angiogenesis-related molecules released in the conditioned media of ES and RMS cells [17].

Nonetheless, *in vitro* studies suggest that an additional mechanism through which HS mimics exert their antitumor activity relies on direct effects on tumor and stromal cells through interference on HSPGs interactions with chemokines, growth factors and receptors. Inhibition of endothelial cell functions stimulated by VEGF and FGF2, such as migration, morphogenesis or proliferation, has been commonly reported as a feature of several HS mimics [46–48]. Among compounds currently undergoing clinical evaluation, the glycol-split heparin M402 (necuparanib) inhibits migration of Jurkat cells induced by the heparin-binding chemokine SDF-1α [49]. The fully sulfated HS mimic PG545 inhibits proliferation of pancreatic tumor cells and migration/invasion of ovarian cancer cells by interacting with Wnts ligands and other heparin binding factors [50, 51]. The recent report showing that roneparstat inhibits chondrogenesis and chondrogenic marker gene expression in mesenchymal cells from mouse embryo [52] suggests a potential therapeutic interest for the drug in treatments of hereditary multiple exostoses, benign pediatric cartilaginous tumors overexpressing heparanase. Of note, interference on signaling mediated by bone morphogenetic proteins, which are well known heparin-binding factors [11, 53] could contribute to the strong anti-chondrogenic effect of roneparstat.

Our present findings support the view of a competition with HSPGs regulatory functions as a relevant mechanism contributing to roneparstat antitumor effects. The formation of a functional ternary complex with FGF family members and FGF receptors has long been known as a characteristic of heparin resembling the co-receptor function of HSPGs [10]. Glycol-split heparin derivatives were shown to maintain the ability to bind bFGF [40]. However, as opposed to heparin, roneparstat induced only a little release of bFGF from the ECM and failed to stimulate its mitogenic activity in early evaluation tests. Present data confirmed that the HS mimic is able to counteract the pro-invasive effect of bFGF and the constitutive activation of FGFR4 and FGFR3. The implication of FGFR4 signaling in RMS tumorigenesis is well documented [54]. The receptor is, in fact, frequently overexpressed through gene amplification or direct transcription by the PAX3-FOXO1 fusion oncoprotein, the hallmark of alveolar RMS. High *FGFR4* expression correlates with advanced stage and poor survival, whereas oncogenic mutations of *FGFR4* are found in a subset of tumors [55]. bFGF, which is abundant in the bone microenvironment, is a main motility factor for ESFT cells [18]. Indeed, expression and activation of FGFRs were observed in clinical samples of ESFT [18] and, in a recent meta-analysis of mutational data, *FGFR3* was found mutated in 50% of ESFT cell lines, being one of the most frequently mutated cancer-associated genes [19].

The IGF/IGF1R/IGFBPs axis represents a nodal signaling system and a potential therapeutic target in both ESFT and OS [20, 56]. In ESFT cells, upregulation of IGF1 and downregulation of the inhibitory IGFBP3 have been described as a direct consequence of the aberrant transcription induced by EWS-FLI1, the fusion oncoprotein pathognomonic of the disease [1, 21, 57]. Moreover, several studies have reported IGF1R expression in ESFT and OS tumor samples and IGF1R signaling dependency in cell cultures [56, 58]. Our data showed inhibition of IGF1R activation in TC71 and U2OS cells exposed to roneparstat and, in agreement, inhibition of colony formation and IGF2-stimulated invasion. Notably, heparin-binding domains are present in four out of six IGFBPs which regulate half-life and bioavailability of IGFs [59, 60]. Specifically, it has been proposed that, by favoring the binding of IGF/IGFBP complexes to “heparin-like glycosaminoglycans” at the cell surface and ECM, certain IGFBPs can increase local IGF activity stimulating nearby IGF1R [59, 60]. In addition, although IGF ligands are not considered canonical heparin-binding proteins, a previously uncharacterized putative heparin-binding domain in IGF2 has been recently demonstrated to play a pivotal role in the IGF2/IGFBP2 complex affinity for heparin [61]. Overall, these studies and our present findings are consistent with interference on the formation of a functional IGF/IGFBP/HS ternary complex as mechanism of inhibition of IGF1R activation by roneparstat.

ERBB4 is an additional target of roneparstat treatment revealed in this study. ERBB4 activation was in fact inhibited by drug treatment in TC71 and SK-N-MC cells which overexpress the receptor [23–25]. ERBB4 expression in ESFT cells has been recently reported to correlate with an aggressive phenotype *in vitro* and *in vivo*. Moreover, in the clinical setting, overexpression of the receptor has been observed in metastatic lesions compared to primary tumors, pointing to a potential role as metastasis biomarker [23]. Our evidence that roneparstat could completely abrogate HB-EGF-induced Matrigel invasion by SK-N-MC cells, support a possible interference of the HS mimic with the interaction between HB-EGF and HSPGs, essential for the ligand function [62, 63]. Indeed, HB-EGF expression and secretion by ESFT and ERMS cells [23 and our data not shown] may sustain activation of both ERBB4 and EGFR. Thereby, the inhibition of ERBB receptors may conceivably rely on roneparstat hampering activation of homo and heterodimers of the ERBB family [28]. Similarly to what observed in ESFT cells, ERBB4 activation was inhibited by roneparstat treatment in the SS cell line CME-1. Notably, also in these cells ERBB4 activation was associated with the cell invasive ability. This finding warrants further investigation since a missense mutation in *ERBB4* has been recently detected, in addition to the t(X;18) main oncogenic driver, in a SS [64]. In SK-N-MC tumor xenografts, ERBB4 and EGFR were the prominent RTKs detected by the phospho-proteome array together with INSR and IGF1R. Their tyrosine phosphorylation was remarkably reduced in roneparstat-treated tumors providing pharmacodynamic evidence of *in vivo* targeting. Notably, decreased angiogenesis was associated with increased apoptosis in tumor cells. These effects are conceivably the result of the multi-targeting heparanase/HS activity of the drug affecting tumor and stromal cells, as well as the ECM.

It is also conceivable that cell-type dependent factors, such as types and HS composition of HSPGs, as well as mechanisms of receptor activation, influence eventual effects of roneparstat on specific signaling pathways. With respect to PDGFRs, consistently with a competition with HSPGs for binding PDGFs, our data support that both autocrine and paracrine modalities of activation can be antagonized by roneparstat. Notably, we demonstrated for the first time the potential of a HS mimic to counteract an oncogenic autocrine loop driving cell transformation in two DFSP model systems, a human primary culture and NIH3T3^COL1A1/PDGFB^ transfectants. Our data clearly indicated that inhibition of the cell membrane pool of constitutively activated PDGFR is associated with inhibition of cell anchorage-independent growth and invading ability by roneparstat. On the other hand, PDGF contributes to the paracrine growth stimulation of different types of stromal cells playing a key role in the cross-talk with malignant cells in the tumor microenvironment. Interference with these PDGF functions may, at least in part, contribute to roneparstat inhibitory effect on angiogenesis observed here, as well as in other studies [15, 43]. It remains to be elucidated whether the PDGF/PDGFR axis inhibition by roneparstat is associated with a reduction of the intratumor fluid pressure, as described for other PDGFR antagonists [31, 65]. The latter effect would be of particular relevance in relation to combination treatments, since a reduced intratumor fluid pressure has been shown to favor drug uptake and therapeutic efficacy [65]. The preclinical profile of roneparstat appears, in fact, especially promising in combination therapies. Previous reports showed good tolerability and potentiation of antitumor efficacy by treatments combining the HS mimic with dexamethasone or other antimyeloma agents, antiangiogenic agents, and lapatinib, in various models [15, 17, 43, 44]. In line with these studies, the combination of roneparstat with the camptothecin irinotecan, was highly effective and well-tolerated in the rhabdoid model A204 being able, in contrast to the singly administered drugs, to block the tumor growth in all treated mice and until the end of the experiment in the majority of animals. These findings implicate a potential clinical interest, since roneparstat is currently under phase 1 evaluation and irinotecan has emerged in pediatric trials as promising for treating RMS and ES in combination therapies [66].

In summary, this study confirms interference with the heparanase/HS functions as a valuable antitumor approach in preclinical sarcoma models and a promising strategy to enhance efficacy in combination therapies. The reported data, demonstrating a multi-target inhibitory effect on activation of coexpressed and often interconnected RTKs crucial in the pathobiology of different sarcoma subtypes, reveals a new aspect, likely cooperating with heparanase inhibition, of the antitumor activity of the HS mimic roneparstat. These findings contribute to provide a preclinical rationale for further investigation in the clinical setting.

## MATERIALS AND METHODS

### Cell lines, culture conditions and drugs

The human rhabdoid A204 and osteosarcoma U2OS cell lines were obtained from American Type Culture Collection, the alveolar RH30 cell line was from Leibniz Institute DSMZ-German Collection of Microorganisms and Cell Cultures. The Askin's tumor cell line SK-N-MC was kindly provided by R. Maggi (University of Milan, Italy), the embryonal RMS cell line RD by A. Rosolen (University of Padua, Italy) and the ES cell line TC71 by M.C. Manara (Rizzoli Institute, Bologna, Italy). The murine NIH3T3 cell line and its derivative NIH3T3^COL1A1/PDGFB^ cell line, obtained by transfection with DNA from a human dermatofibrosarcoma protuberans containing the COL1A1/PDGFB rearrangement [5] were kindly provided by A. Greco (Fondazione IRCCS Istituto Tumori, Milan, Italy). The SS cell line CME-1 was previously described [67]. The short term culture of DFSP was obtained by enzymatic disaggregation of a fresh specimen. Patient provided written consent for the use of specimen for research.

RD, RH30, A204 and CME-1 cells were cultured in RPMI medium, TC71 cells in Iscove's modified Dulbecco's medium, U2OS cells in McCoys medium, and SK-N-MC cells in EMEM medium (Lonza, Verviers, Belgium). The DSFP-C primary culture was maintained in DMEM:Ham's F12 (1:1) medium (Lonza). Culture media were supplemented with 10% fetal bovine serum. The above listed cell lines were maintained at 37°C in a 5% CO_2_ atmosphere. NIH3T3 and NIH3T3^COL1A1/PDGFB^ fibroblasts were cultivated in DMEM supplemented with 10% or 5% calf serum, respectively (Colorado Serum Company, Denver, CO) in a 10% CO_2_ atmosphere. All tumor cell lines were authenticated by the AmpFISTR Identifiler PCR amplification kit (Applied Biosystems, PN4322288) and only frozen pools of tested cells were used.

Roneparstat (SST0001) was provided by sigma-tau Research Switzerland S.A. (Mendrisio, CH). Preparation and characterization of roneparstat, characterized by N-acetylation and glycol-splitting (previously known as ^100^NA-ROH), have been previously reported [40]. Roneparstat was dissolved in physiological saline and irinotecan in distilled water.

### Antibodies

Mouse monoclonal antibodies: anti-phospho-FAK(Tyr397) from BD Transduction (Lexington, KY); anti-EGFR and anti-phosphotyrosine clone 4G10, from Upstate Biotechnology (Lake Placid, NY); anti-β tubulin and anti-ERBB3 from Sigma-Aldrich (St. Louis MO); anti IGF-IRβ from Santa Cruz Biotechnology (Santa Cruz, CA). Rabbit polyclonal antibodies: anti-actin and anti-ERBB4 from Sigma; anti-phospho-FGFR (Tyr653/654), anti-phospho-HER2/ERBB2 (Tyr877), anti-phospho-HER4/ERBB4 (Tyr984) and anti-FAK from Cell Signaling (Beverly, MA); anti-PDGFR β and anti-PDGFRα from Upstate Biotechnology; anti-FGFR3 and anti-FGFR4 from Santa Cruz Biotechnology. Rabbit monoclonal antibodies: anti-phospho-PDGFRα (Tyr849)/PDGFRβ (Tyr857), anti-phospho-IGF-IRβ (Tyr1135/1136)/InsulinRβ (Tyr1150/1151), anti-phospho-HER3/ErbB3 (Tyr1289), anti-PDGFRβ and anti-HER2/ERBB2 from Cell Signaling.

### Cellular studies

For the cell growth inhibition assay, cells were plated at 2500 cells/cm^2^, treated the day after with the indicated drug concentrations and counted 72 h later using a Coulter Counter (Coulter Electronics, Luton, UK). For the anchorage-independent growth assay in soft agar, the previously described procedure was applied [68]. Briefly, cells, seeded at 500–1000 cells/cm^2^, were incubated in the presence of solvent or drug for 11–26 days then, colonies were counted under a magnifying projector. Alternatively, the number of colonies in six fields was counted for each plate. The size and the number of colonies were determined by ImageMaster TotalLab, version 1.10, analyzing digital images captured by Image Master VDS (Amersham Biosciences Little Chalfont, UK). For the Matrigel invasion assay, cells were seeded in complete medium and pretreated at the indicated drug concentrations for 24 h. Then, cells were harvested, resuspended in serum-free or in the cells' own conditioned medium as indicated, and transferred to the upper chamber of 24-well Transwell plates (Costar, Corning Inc., Corning, NY) previously coated with Growth Factor Reduced Matrigel (BD Biosciences, San Jose, CA) (6 × 10^4^ – 2,4 × 10^5^ cells/filter, according to the spontaneous invasive ability). The same drug concentration used for cell pretreatment was added in both the upper and lower chambers. Where indicated, human recombinant growth factors were added in the lower chamber at 50 ng/ml. After 24 h (or 48 h for experiments with the SK-N-MC cell line), cells that invaded Matrigel were stained with sulforodhamine B (SRB) (Sigma) and counted under an inverted microscope as described [17]. The number of invading cells in four microscopic fields was counted for each filter. The following human recombinant growth factors were used: HGF, HB-EGF, PDGFAA and PDGFBB from Sigma, basic FGF and EGF from Peprotech (London, UK), IGF2 from R&D system (Minneapolis, MN).

### Western blot analysis and RTK proteome profiler

For biochemical analyses, exponentially growing cells were seeded in complete medium and treated the day after with the drug at the indicated concentrations. After 48h, cells were processed for RTK analysis using the Proteome Profiler Array Kit (ARY001/ARY001B, R&D systems) according to the manufacturer's instructions, or for total protein extraction, or immunoprecipitation as previously described in details [68, 69]. Otherwise, proteomic analysis was performed on lysates from serum starved cells, or on lysates from frozen tumors analogously processed after pulverization by the Mikro-Dismembrator II (B. Brown Biotech International, Melsungen, Germany). For validation experiments, immunoprecipitates or cell lysates were prepared, separated by SDS-PAGE, transferred on nitrocellulose and analyzed by western blotting as described [68], using the indicated antibodies.

### Immunofluorescence analyses

Cells, grown on coverslips, were exposed to the drug for 48 h, then fixed in 3% paraformaldehyde for 15 min and permeabilized in cold 100% methanol for 1 min. After blocking in 1% BSA in PBS for 1 h and washing in PBS, cells were incubated with primary anti-phospho-PDGFR antibody (1:500) followed by secondary Alexa Fluor 488 anti-rabbit antibody (Invitrogen, Carlsbad, CA). Alternatively, cells were fixed in 3.7% formaldehyde for 20 min and permeabilized with 0.2% Triton X-100 in PBS for 5 min at room temperature. After blocking with 2% BSA in PBS, slides were incubated with Fluorescein Isothiocyanate Labeled Phalloidin (1:500) (Sigma). Nuclei were counterstained with Hoechst 33341 (Sigma). Slides, mounted with Mowiol, were examined by a fluorescence microscope equipped with a digital camera.

### Fluorescent *In Situ* Hybridization

*COL1A1* and *PDGFB* gene status were studied by FISH on cells from primary culture by using Bacterial artificial chromosome (BAC) probes (Children Hospital Research Institute, Oakland, CA) covering the *PDGFB* (RP11-630N12 RP11-506F7) and *COL1A1* (RP11-93L18, RP11- 131M15) genes [70]. BACs were labelled with Spectrum Green or Spectrum Orange (Abbott Molecular, Abbott Park, IL) by nick translation (Nick translation KIT; Abbott Molecular). Probe labelling and cells treatments for FISH were carried out according to manufacturer's instruction.

### *In vivo* studies

All experiments were carried out using 8 weeks-old female athymic Swiss nude mice (Charles River, Calco, Italy). Mice were maintained in laminar flow rooms keeping temperature and constant humidity with free access to food and water. Experiments were approved by the Ethics Committee for Animal Experimentation of the Fondazione IRCCS Istituto Nazionale dei Tumori of Milan according to reported guidelines [71].

Cells exponentially growing in cell culture were injected s.c. in mice and tumor lines were achieved by serial s.c. passages of tumor fragments. For antitumor activity studies, fragments from growing tumors were s.c. implanted in the right flank of mice. Groups of 7–8 mice bearing one tumor s.c. were employed. Treatments started 1–3 days after the engraftment. Roneparstat was administered s.c., twice daily (60 mg/kg/injection), for 6 consecutive days per week (2qdx6/w), with the treatment repeated for 4–6 weeks. Irinotecan was administered i.v. (q4dx4) at 50 mg/Kg. The efficacy of drug treatments was assessed as: tumor volume inhibition percentage (TVI %) in treated versus control mice, calculated according to the formula: TVI% = 100 − (mean TV treated/mean TV control × 100); complete regression (CR), i.e. disappearance of the tumor lasting at least ten days after the end of treatments; no evidence of disease (NED), i.e. absence of tumors at the end of the experiment. Drug tolerability was assessed as body weight loss percent which never exceeded 10% during treatments.

For histological and immunohistochemical analyses, mice carrying s.c. SK-N-MC xenografts (three to five mice per group) were treated with roneparstat at the dose 60 mg/kg (2qdx5/w). After 16 days from the beginning of treatment, tumors were excised, formalin fixed and paraffin embedded. Four μm sections from each tumor xenograft were stained with Hematoxylin-Eosin and the number of mitoses and apoptosis was morphologically assessed in 3 randomly selected high power fields within the tumor section. Microvessel density was evaluated by immunohistochemical detection of CD31 using a primary rat monoclonal antibody (Dianova, Hamburg, Germany). The number of CD31-positive vascular outlines was counted in 3 200x microscopic fields randomly selected throughout the neoplastic tissue by using the ImageJ analysis program. Histopathological and immunohistochemical analyses were performed in a blind fashion.

For *in vivo* pharmacodynamic evaluation, two hours after the last drug administration, the animals were sacrificed and the tumors were resected and snap frozen in liquid nitrogen before processing for RTK proteomic profiling.

### Statistical analyses

The Student's 2-tailed *t* test was applied to assess statistical significance in *in vitro* and *in vivo* experiments. *P* values < 0.05 were considered significant.

## SUPPLEMENTARY MATERIALS AND FIGURES


