# Supplementary material for: Antitumor efficacy of the heparan sulfate mimic roneparstat (SST0001) against sarcoma models involves multi-target inhibition of receptor tyrosine kinases
Source: Oncotarget. 2016 Jun 25;7(30):47848–63. doi: 10.18632/oncotarget.10292 (PMC5216983; doi:10.18632/oncotarget.10292)
Supplement: Supplementary file 1 [file oncotarget-07-47848-s001.pdf]

Antitumor efficacy of the heparan sulfate mimic roneparstat (SST0001) against sarcoma models involves multi-target inhibition of receptor tyrosine kinases

Supplementary Materials

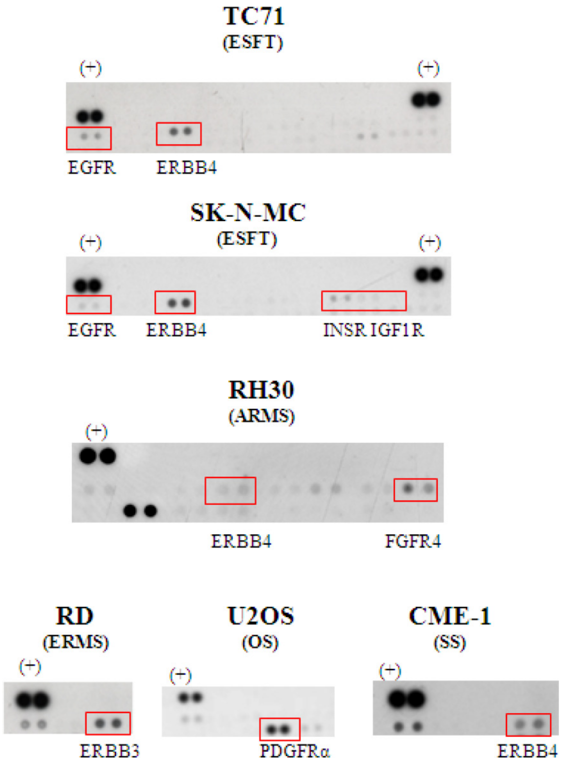

Supplementary Figure S1: Proteomic profiling showing constitutive activation of selected RTKs in sarcoma cell lines. The day after cell seeding, complete medium was removed and, after washing with saline, cells were incubated in serum-free medium for 48 h. Then, cells were lysed and processed for analysis with human phospho-RTK array. Rectangles evidence RTKs investigated in this study. (+), reference spots. ESFT, Ewing's sarcoma family tumors; ARMS, alveolar rhabdomyosarcoma; ERMS, embryonal rhabdomyosarcoma; OS, osteosarcoma; SS, synovial sarcoma.

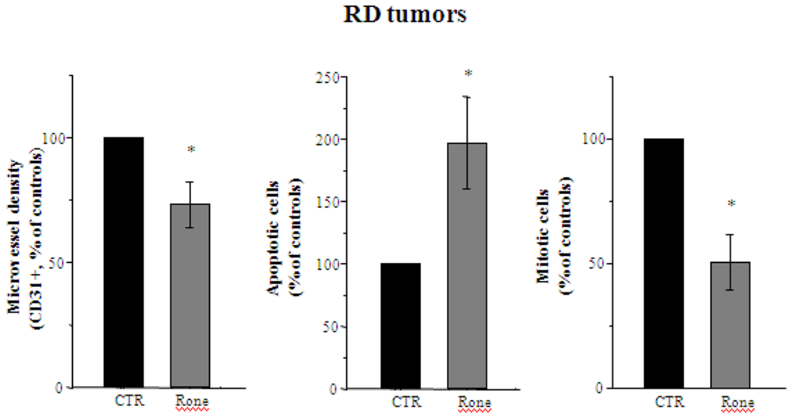

Supplementary Figure S2: Reduction of microvessel density and mitoses, and increased apoptosis in ERMS RD tumors from mice administered with roneparstat. Microvessel density was assessed on tumor sections stained immunohistochemically for CD31, a specific marker of endothelial cells. Tumors were sampled from mice receiving vehicle (CTR) or drug (Rone) at 60 mg/kg, 2qdx5/w, for 3 weeks. Morphological detection of mitotic and apoptotic cells was performed on Hematoxylin-Eosin stained sections from mice administered with roneparstat at 60 mg/kg, 2qdx5/w, for 4 weeks. Columns, mean percentage of controls ± SE. *$P < 0.05$.
